# Endogenous phospholipase A_2_ inhibitors in snakes: a brief overview

**DOI:** 10.1186/s40409-016-0092-5

**Published:** 2016-12-21

**Authors:** Patrícia Cota Campos, Lutiana Amaral de Melo, Gabriel Latorre Fortes Dias, Consuelo Latorre Fortes-Dias

**Affiliations:** Laboratory of Enzymology, Center of Research and Development, Ezequiel Dias Foundation (FUNED), Rua Conde Pereira Carneiro, 80, Gameleira, Belo Horizonte, MG CEP 30510-010 Brazil

**Keywords:** PLA_2_ inhibitor, Phospholipase A_2_, Snake blood, Natural resistance, Snakes

## Abstract

The blood plasma of numerous snake species naturally comprises endogenous phospholipase A_2_ inhibitors, which primarily neutralize toxic phospholipases A_2_ that may eventually reach their circulation. This inhibitor type is generally known as snake blood phospholipase A_2_ inhibitors (sbPLIs). Most, if not all sbPLIs are oligomeric glycosylated proteins, although the carbohydrate moiety may not be essential for PLA_2_ inhibition in every case. The presently known sbPLIs belong to one of three structural classes – namely sbαPLI, sbβPLI or sbγPLI – depending on the presence of characteristic C-type lectin-like domains, leucine-rich repeats or three-finger motifs, respectively. Currently, the most numerous inhibitors described in the literature are sbαPLIs and sbγPLIs, whereas sbβPLIs are rare. When the target PLA_2_ is a Lys49 homolog or an Asp49 myotoxin, the sbPLI is denominated a myotoxin inhibitor protein (MIP). In this brief overview, the most relevant data on sbPLIs will be presented. Representative examples of sbαPLIs and sbγPLIs from two Old World – *Gloydius brevicaudus* and *Malayopython reticulatus* – and two New World – *Bothrops alternatus* and *Crotalus durissus terrificus* – snake species will be emphasized.

## Background

A number of venomous and nonvenomous snake species are naturally resistant to the deleterious actions of snake venom components, in many cases due to the presence of specific antitoxins in their circulating blood [1–10]. These antitoxins were identified as liver-secreted proteins, which prevent any possible damage from toxins that might have reached the snake’s blood stream [11]. Among these inhibitors, phospholipase A_2_ inhibitors or snake blood phospholipase A_2_ inhibitors (sbPLIs) play a key role in this type of endogenous resistance.

During the 80’s and 90’s, a number of sbPLIs were purified from different snake species. The first authors to identify various sbPLIs in a single snake species – *Gloydius brevicaudus*, formerly *Agkistrodon blomhoffii siniticus* – proposed a classification based on the presence of characteristic domains of known mammalian proteins in their structure and on variations in their PLA_2_ selectivity [12]. Alpha sbPLIs (sbαPLIs) have a C-type lectin-like domain that is highly similar to the carbohydrate recognition domain of Ca^2+^-dependent lectins, and preferentially inhibit acidic PLA_2_s. Beta-type inhibitors (sbβPLIs) exhibit tandem leucine-rich repeats (LRRs), and specifically inhibit basic PLA_2_s. Gamma inhibitors (sbγPLIs) display a three-finger pattern and are less specific than the aforementioned classes, therefore inhibiting neutral, acidic and basic PLA_2_s from snake venoms. The structural classification of sbPLIs has been adopted by most authors working on the subject, but the selectivity concept is not absolute [13–16]. In general, α and γ sbPLIs simultaneously occur in several snake species, while sbβPLIs have only been reported in three snake species.

Native sbPLIs are usually homo- or heterooligomers of glycosylated and/or non-glycosylated subunits. Carbohydrates do not seem essential for the inhibition of PLA_2_ by sbPLIs, since some of them remain functional in the absence of this moiety [16–20]. When the target PLA_2_s are Lys49 homologues or Asp49 myotoxins, the sbPLIs are specifically called myotoxin inhibitor proteins (MIPs) [13, 14, 16, 21, 22].

The following sections present the most relevant characteristics of the three classes of sbPLIs. Subsequently, examples of sbαPLIs and sbγPLIs from two Old World snake species — *Gloydius brevicaudus* and *Malayopython reticulatus* — and two New World ones — *Bothrops alternatus* and *Crotalus durissus terrificus* — will be introduced.

## Alpha class of sbPLIs (sbαPLIs)

Members of this class of inhibitors are found in solution as homo- or heterooligomers, with molecular masses between 75 kDa and 120 kDa (Table 1).

**Table 1:** Snake blood PLA_2_ inhibitors in the alpha structural class (sbαPLIs)

Family, species or subspecies	Common name	Reference
Colubridae		
*Elaphe climacophora*	Japanese ratsnake	[26]
*Elaphe quadrivirgata*	Japanese four-lined ratsnake	[27]
Viperidae		
*Bothrops alternatus*	*Urutu* (Portuguese)	[15, 31]^a^, [23]
*Bothrops asper*	*Fer-de-lance, Terciopelo* (Spanish)	[62]
*Bothrops erythromelas*	Caatinga lancehead	[23]
*Bothrops jararaca*	*Jararaca* (Port.)	[23]
*Bothrops jararacussu*	*Jararacussu* (Port.)	[14, 23]
*Bothrops moojeni*	Brazilian lancehead	[16]
*Bothrops neuwiedi*	*Jararaca pintada* (Port.)	[23]
*Cerrophidion godmani*	Honduras montane pit viper	[21]
*Crotalus durissus terrificus*	South American rattlesnake, tropical rattlesnake	[23]
*Gloydius brevicaudus*	Short-tailed mamushi, Japanese or Chinese mamushi,	[19]^a^, [63]
*Lachesis muta*	South American bushmaster	[23]
*Protobothrops flavoviridis*	Habu	[25, 64, 65], [66]^a^
*Protobothrops elegans*	Sakishima habu	[41]

^a^Recombinant homolog

In addition to the typical C-type lectin-like domain, sbαPLI monomers present two other highly conserved regions in their structure: a hydrophobic core at their carboxy-terminus and an α-helical coiled-coil neck comprising the 13^th^ to 36^th^ amino acid segment in the mature protein [23, 24]. The last amino acid stretch corresponds to the exon 3 reported for the gene of the sbPLI from *Protobothrops flavovoridis* (formerly *Trimeresurus flavoviridis*) [25].

Besides the functional sbαPLIs, non-functional homologs were purified from the blood serum of two nonvenomous species, *Elaphe quadrivirgata* and *E. climacophora*. Despite displaying not only molecular masses, but also primary and quaternary structures comparable to classical sbαPLIs, these homologs failed to inhibit all tested snake venom PLA_2_s [26, 27].

### The sbαPLI from Asian *Gloydius brevicaudus* (GbαPLI)

The sbαPLI from *G. brevicaudus* (formerly *Agkistrodon blomhoffii siniticus*) is a homotrimer, in which the α-helical coiled-coil neck subunit forms a central pore that constitutes the binding site for the target PLA_2_s [28–30]. The C-type lectin-like domain was discarded as responsible for PLA_2_ binding [30].

The correct configuration of the central pore in GbαPLI is controlled by the primary structures of the α-helical coiled-coil neck in the formation of subunits. Chimeric constructions of GbαPLI and the non-functional sbαPLI homolog from *E. quadrivirgata* allowed the mapping of important amino acids for PLA_2_ inhibition in the 13–36 segment, which are expected to be located in the helical neck of the GbαPLI trimer based on the three-dimensional structural model constructed by homology modeling [29, 30]. The trimerization occurs only among subunits having the same α-helical motif in the regions 13–36 and the oligomer is structurally stabilized by intermolecular electrostatic interactions. Two charged residues, E^23^ and K^28^, have been found specifically responsible for these essential interactions between the forming subunits in the trimer. The contribution of each subunit to the total inhibitory activity of trimeric GbαPLI has also been investigated. In the trimer, the inhibitory action is driven by one subunit with the highest affinity and is not affected by the number of subunits of this type [29].

GbαPLI displays lower affinities (about 2000-fold less) for neutral or basic PLA_2_s from the homologous venom compared to acidic PLA_2_s. In the absence of carbohydrates, the inhibition of acidic and neutral PLA_2_s has been reported to remain unchanged, while the inhibition of basic PLA_2_s is affected [19]. The possibility of different inhibition mechanisms, depending on the ionic character of the target PLA_2_, has been attributed to GbαPLI and other sbαPLIs, but further studies are required to clarify this issue.

### The sbαPLI from Latin American *Bothrops alternatus* (BaltMIP)

This inhibitor was purified from the blood serum of *Bothrops alternatus* snakes by affinity chromatography using bothropstoxin I – a basic Lys49 PLA_2_ from the homologous venom – as the immobilized ligand. The monomer of BaltMIP is composed of a single polypeptide chain with apparent molecular mass of 24 kDa. The native molecule is able to inhibit myotoxicity and cytotoxicity caused by both Lys49 and Asp49 PLA_2_s, possibly by different mechanisms depending on the type of enzyme to be inhibited [15]. Amino acid residues possibly involved in the inhibition by BaltMIP of acidic PLA_2_s from homologous venom have been recently discussed in comparison to published data for PLA_2_-sbαPLIs complexes from Asian snake species [23].

The characteristic α-helical coiled-coil neck, the carbohydrate recognition domain and the hydrophobic core of sbαPLIs are well conserved in the BaltMIP monomer, according to the theoretical structural model (available in the Model Archive database under DOI: 105452/ma-a2iil). In the trimeric BaltMIP (available in the Model Archive database under DOI: 105452/ma-a4btt), three monomers fit well in a spherical arrangement [15].

Recombinant BaltMIP, displaying the same apparent molecular mass (24 kDa) as the native inhibitor monomer, has been produced in *Pichia pastoris*. The expressed protein was heavily glycosylated and formed oligomers of about 77 kDa, a profile fully compatible with a trimeric arrangement. Nevertheless, the functionality of the recombinant protein was reduced in comparison with the native molecule [31].

## Beta class of sbPLIs (sbβPLIs)

Beta-type inhibitors are acidic, leucine-rich glycoproteins of 150–160 kDa. The leucines are assembled as leucine-rich repeats (LRRs) in tandem. This particular arrangement creates horseshoe-shaped molecules, similarly to those observed in Toll-like receptors in general [12, 32]. The first sbβPLI described in the literature was purified from *G. brevicaudus* as a homotrimer (Table 2). The inhibitor is specific for basic PLA_2_s from homologous venom and forms a stable PLA_2_-sbβPLI complex at a 1:1 molar ratio [12, 33].

**Table 2:** Snake blood PLA_2_ inhibitors in the structural beta class (sbβPLIs)

Family, species or subspecies	Common name	Reference
Colubridae		
*Elaphe climacophora*	Japanese ratsnake	[26]
*Elaphe quadrivirgata*	Japanese four-lined ratsnake	[34]
Viperidae		
*Gloydius brevicaudus*	Short-tailed mamushi, Japanese or Chinese mamushi	[33]
*Lachesis muta*	South American bushmaster	[67]

Subsequently, similar sbβPLIs were purified from two non-venomous Colubridae snakes: *E. quadrivirgata* and *E. climacophora* [26, 34] (Table 2). Besides nine LRRs of 24 amino acids each, all three known sbβPLIs display a proline-rich amino-terminal region and ten cysteines, eight of which are probably involved in disulfide bonds. The fully conserved LRR1 segment might be responsible for the specific binding of sbβPLIs to basic PLA_2_s [26].

## Gamma class of sbPLIs (sbγPLIs)

Currently, the gamma class of phospholipase A_2_ inhibitors comprises the greatest number of endogenous sbPLIs (Table 3).

**Table 3:** Snake blood PLA_2_ inhibitors in the structural gamma class (sbγPLIs)

Family, species or subspecies	Common name	Reference
Colubridae		
*Elaphe climacophora*	Japanese ratsnake	[26]
*Elaphe quadrivirgata*	Japanese four-lined ratsnake	[68]
*Sinonatrix annularis*	Ringed water snake	[69]^a^
Elapidae		
*Naja naja kaouthia*	Monocled cobra, Thailand cobra	[35]
*Notechis ater*	Tasmanian tiger	[70]
*Notechis scutatus*	Mainland tiger snake, common tiger snake	[37]
*Oxyuranus microlepidotus*	Fierce snake, Inland taipan	[42]
*Oxyuranus scutellatus*	Coastal taipan, New Guinea taipan	[42]
*Pseudonaja textilis*	Eastern brown snake, common brown snake	[42]
Pythonidae		
*Python reticulatus*	Reticulated python	[20]^a^
*Python sebae*	African python	[50]
Viperidae		
*Bothrops alternatus*	*Urutu* (Portuguese)	[61]
*Bothrops erythromelas*	Caatinga lancehead	[61]
*Bothrops jararaca*	*Jararaca* (Port.)	[61]
*Bothrops jararacussu*	*Jararacussu* (Port.)	[22], [61]
*Bothrops neuwiedi*	*Jararaca pintada* (Port.)	[61]
*Cerrophidion godmani*	Honduras montane pit viper	[21]
*Crotalus durissus collilineatus*	Brazilian rattlesnake	[71]
*Crotalus durissus terrificus*	South American rattlesnake, tropical rattlesnake	[51–53]
*Lachesis muta*	South American bushmaster	[72]
*Gloydius brevicaudus*	Short-tailed mamushi, Japanese mamushi or Chinese mamushi	[73]
*Protobothrops flavoviridis*	Habu	[25]
*Protobothrops elegans*	Sakishima habu	[41]

^a^Recombinant homolog

SbγPLIs are acidic glycoproteins characterized by two structural units of highly conserved repeats of half cysteines, known as three-finger motifs, such as those found in proteins belonging to the Ly-6 family, the urokinase-type plasminogen activator, and α-neurotoxins [35, 36]. A subclassification into classes 1 and 2 was subsequently proposed for sbγPLIs, based on predicted structural homologies to urokinase-type plasminogen activator receptor (u-PAR) or to Ly-6. The inhibitors with the highest homology to the u-PAR were located in class 1, whereas those more similar to Ly-6 were assigned to class 2 [37].

Another important characteristic of most sbγPLIs is a highly conserved proline-rich region [38]. Proline residues are commonly found in the flanking segments of protein–protein interaction sites. Known as proline brackets, they may play a structural role by protecting the integrity and conformation of the interaction sites in functional proteins [39].

SbγPLIs may be assembled as hetero- or homomeric molecules and a subclassification was proposed based on the monomer composition [40]. The sbγPLIs from elapids (*Naja naja kaouthia, Notechis ater, Notechis scutatus* and *Oxyuranus scutellatus*), colubrid (*Elaphe quadrivirgata)*, Old World viperid (*Gloydius brevicaudus)* and hydrophiid (*Laticauda semifasciata)* were placed in subclass I (heteromeric). All these inhibitors are composed of two different subunits with distinct primary structures (called α and β, or A and B) typically under a 2:1 ratio for A and B, respectively.

Subclass II is comprised of homomeric sbγPLIs from New World viperid *Bothrops asper*, *Cerrophidion godmani*, and *C. d. terrificus*, as well as *Malayopyton reticulatus* (Pythonidae) and *P. flavovirids* (Viperidae) from the Old World. However, the identification of a secondary subunit, similar to the subunit B of heteromeric inhibitors, in the sbγPLI-IIs from *C. d. terrificus*, *P. elegans P. flavoviridis* and several Australian elapid species challenged the homomeric composition of those inhibitors [17, 41–43]. However, a single subunit remained in sbγPLI-IIs from *M. reticulatus*, *C. godmani,* and *B. jararacussu*. The last two were originally purified by affinity chromatography using the target PLA_2_s as an immobilized ligand, whereas the purified inhibitors were confirmed as being composed of single subunits A, as expected for sbγPLI-IIs. All three sbγPLI-IIs were fully functional as homomers [21, 22]. The actual contribution of the secondary subunits B to the full functionality of the sbγPLI-IIs, whenever applicable, remains to be clarified. It has been speculated that the subunit B might play a structural rather than a functional role in the sbγPLIs from Australian elapid species [42]. On the other hand, an ancestral role has been suggested for the subunit B compared to subunit A, in the sbγPLI from the Asian *P. flavoviridis* [43]. In any case, both subunits, A and B, may be present as a heterogeneous mixture of more and less conserved isoforms, therefore generating subtle structural changes depending on the combination of isoforms, and increasing the PLA_2_-binding repertoire of sbγPLIs [38, 41].

### The sbγPLI from Asian *Malayopython reticulatus*

This inhibitor was denominated phospholipase inhibitor from python (PIP). The native protein is a glycosylated oligomer formed by six identical subunits of 23 kDa each. After full deglycosylation, the molecular mass of the subunits decreases to 20 kDa.

Native PIPs occur as hexamers of apparent molecular mass of 140 kDa. The monomer precursor in snake liver tissue has a 19-residue signal sequence and an open reading frame of 603 bp encoding for a 182-residue protein. PIPs neutralize both lethal and PLA_2_ activities of daboiatoxin – the major toxin of *Daboia russelli siamensis* snake venom – by forming a toxin-inhibitor complex at 1:1 molar ratio. A recombinant PIP homologue produced in *Escherichia coli* was shown to neutralize not only daboiatoxin PLA_2_ activity in vitro*,* but other toxic PLA_2_s belonging to groups I (from Elapidae snake venoms), II (from Viperidae snake venoms) and III (from bee venom) at inhibitor-enzyme molar ratios between 0.1 and 5.0. In addition, this PIP homolog inhibited the edematogenicity of bee venom PLA_2_ and daboiatoxin up to 92.1 and 78.2%, respectively [20].

The functional site of PIP was predicted based on the hypothesis of proline brackets, and the data were employed to design PIP-derived bioactive peptides [39]. In general, the inhibition of PLA_2_s by these peptides has been explained by the blockage of the hydrophobic channel of secreted PLA_2_, as presented by other known inhibitors of this enzyme type [44].

Among a number of linear and cyclic PIP-derived peptides tested, PGLPPLSLQNG decapeptide (called P-PB.III) was able to inhibit groups I, II and III of PLA_2_s, including PLA_2_ from human synovial fluid of arthritis patients belonging to subgroup IIA [45]. The heptadecapeptide LGRVDIHVWDGVYIRGR (named PNT.II) was found to selectively inhibit human secreted IIA-PLA_2_. It also reduces neurotoxin-induced high levels of secreted PLA_2_ in rat hippocampal homogenates and modulates joint destruction in a mouse model of human rheumatoid arthritis [44, 46, 47]. An analog of PNT.II, known as PIP18, has been more recently devised [48]. Besides potent neutralization effects against *Crotalus adamanteus* snake venom, PIP18 has shown high bactericidal action against a number of pathogens, in a dose-dependent manner, with a remarkable activity against *Staphylococcus aureus.* Topical application of PIP18 has also modulated in vivo wound repair in a mouse model of *S. aureus* infection [49].

A structurally-related PIP homolog was later isolated from another pythonid species, *Python sebae*. Despite displaying poor PLA_2_ inhibition activity, the primary structure is highly similar to that of PIP. Two subunits (A and B) were characterized in this novel molecule. Nevertheless, both of them display the same amino-terminal sequence and show no similarity with the previously described B subunits from typical heteromeric sbγPLI-Is [50]. As to the complete primary structure, subunits A and B in the PIP homolog differ in eight of 182 amino acids, which suggests that they are actually isoforms of subunit A. Henceforth, the homomeric character of PIP appears preserved in PIP homolog.

### The sbγPLI from Latin American *Crotalus durissus terrificus*

The cDNA of *C. d. terrificus* sbγPLI – called *Crotalus* neutralizing factor (CNF) – encodes a 19-residue signal peptide characteristic of secreted proteins, followed by 181 amino acids in the mature protein, including sixteen cysteines. CNF is a glycosylated alpha_1_-globulin with a single N-linked carbohydrate site at Asn^157^ [51–54]. The carbohydrate moiety, however, is not essential for PLA_2_ inhibition, since CNF remains functional after enzymatic deglycosylation [17].

Native CNF is a globular-shaped, predominantly tetrameric molecule with an average molecular mass of 100 kDa in solution. It innately occurs as a mixture of non-glycosylated and glycosylated monomers of 22 kDa and 25 kDa, respectively [55]. The oligomerization of CNF is independent of the presence of carbohydrates, since it occurs equally with native or enzymatically deglycosylated monomers. Tyrosine residues at the interface of the monomers composing CNF may contribute to the oligomerization process, according to a theoretical structural model constructed for the inhibitor (available with DOI:10.5452/ma-avb44 at ModelArchive database). The U monomer of the crystallographic structure of urokinase plasminogen activator from *Homo sapiens* (PDB ID: 2FD6) was used as the template *ab initio* [17].

Besides inhibiting lethal and PLA_2_ actions of *C. d. terrificus* venom, CNF is also able to inhibit the lethal activity of heterologous viperid venoms, such as those from *Bothrops alternatus, B. atrox, B. jararaca. B. jararacussu, B. moojeni, B. neuwiedi* and *Lachesis muta*, but not that of the elapid *Micrurus frontalis* [51]. In relation to PLA_2_ inhibition of heterologous venoms, CNF is capable of fully inhibiting the PLA_2_ activity of crude venom and of a semi-purified fraction of *L. muta,* which comprise PLA_2_s of different ionic character*.* It is important to note that the crude venom of *L. muta* is about eight times more active than *C. d. terrificus* venom, whereas the aforementioned fraction displays even higher activity – almost 24 times higher than that of *C. d. terrificus* venom [56]. Soon after, investigations of a protein highly similar to CNF purified from *C. d. terrificus* serum, named crotoxin inhibitor from *Crotalus* serum (CICS) by Perales and co-workers, showed complex formation with monomeric and multimeric Viperidae β-neurotoxins [53]. The enzymatic activity of pancreatic and non-pancreatic PLA_2_ from mammals, bee venom and Elapidae venoms remained unaffected [57].

The natural target of CNF in homologous venom is crotoxin, a heterodimeric β-neurotoxin formed by an enzymatically inactive subunit (crotoxin A or CA) and a PLA_2_ counterpart (crotoxin B or CB). CA and CB are non-covalently bonded in the crotoxin complex (CA/CB) [58]. CNF is able to displace CA in the native crotoxin in vitro to form a non-toxic CNF/CB complex, most likely at a 1:1 molar ratio [52]. In the presence of CNF, the newly formed CNF/CB complex no longer interacts with the target acceptor of crotoxin on rat brain synaptosomes to deliver CB to cause its toxic effect [55]. The formation of a new nontoxic complex by displacement of CA from the native crotoxin was confirmed by studies on the interaction of CICS and heterodimeric β-neurotoxins (Mojave toxin, CbICbII from *Pseudocerastes fieldi* venom, and crotoxin itself) [57].

The interaction in CNF/CB complex may be reminiscent of the crotoxin-receptor interaction at the presynaptic site. Competitive binding experiments were performed in vitro on rat brain synaptosomes, in an attempt to clarify the role of CNF as a CB receptor. Although the model encompasses a unique mechanism with three molecules – the receptor on synaptosomal membrane, CNF, and CA – competing for one ligand (CB), comparable IC_50_ values of around 100 nM were found for both CNF and CA [55].

It has been suggested that amino-terminus, beta-wing and carboxyl-terminus regions of CB participate in the formation of CA/CB and CNF/CB complexes [59]. The counter segments in CA and CNF remain to be clarified. A screening of highly conserved regions in CNF and putative sbγPLIs from Latin American pit vipers, using the multiple EM for motif elicitation (MEME) software for multiple alignment [60] indicated the decapentapeptide QPFPGLPLSRPNGYY as the best consensus motif possibly involved in the PLA_2_ interaction. Compared to the aforementioned decapeptide P-PBIII from PIP, the motif displays an amino-acid deletion and two amino-acid substitutions in its internal decapeptide [61].

## Conclusion

A brief review on past and recent achievements on sbPLIs is presented herein. Although they have been studied by different groups over the years, many gaps remain to be filled, especially concerning their action mechanism and scope. In the near future, a better understanding of sbPLIs may guide practical applications of these fascinating molecules in biotechnology and therapeutics on PLA_2_-related disorders.

## Abbreviations

BaltMIP: Myotoxin inhibitor protein from *Bothrops alternatus*
CA: Crotoxin A
CB: Crotoxin B
CICS: Crotoxin inhibitor from *Crotalus* serum
CNF: *Crotalus* neutralizing factor
GbPLI: Phospholipase A_2_ inhibitor from *Gloydius brevicaudus*
LRR: Leucine-rich repeats
MEME: Multiple EM for motif elicitation
MIP: Myotoxin inhibitor protein
PIP: Phospholipase A_2_ inhibitor from *Python*
PLA_2_: Phospholipase A_2_
sbPLI: Snake blood phospholipase inhibitor
u-PAR: urokinase-type plasminogen activator receptor
